# Development and validation of a 28-day mortality risk prediction model for patients with AIDS suffering from sepsis secondary to pulmonary infections in the ICU

**DOI:** 10.1186/s12879-026-13108-w

**Published:** 2026-03-18

**Authors:** Qihui Ran, Xuan Zhao, Qing Du, Chao Deng, Jing Gan, Dingshan Zhang, Hong Chen

**Affiliations:** Department of Critical Care Medicine, Public Health Clinical Center of Chengdu, 377 Jingming Road, Chengdu, Sichuan 610061 China

**Keywords:** Sepsis, AIDS, Pulmonary infection, Mortality, Prediction model

## Abstract

**Background:**

Patients with AIDS Suffering from sepsis secondary to pulmonary infections admitted to intensive care units (ICUs) carry a high burden of morbidity and mortality. Therefore, rapid identification and timely clinical decision-making are essential to optimize patient outcomes.

**Methods:**

Patients with AIDS and sepsis secondary to pulmonary infections admitted to the ICU of Chengdu Public Health Clinical Medical Center (January 2016–June 2025) were stratified into a development cohort (*n* = 385) and a temporal validation cohort (*n* = 86). Clinical and laboratory variables within the first 24 h of ICU admission were retrospectively extracted; for those with repeated measurements, the values reflecting the most severe clinical status were selected. Univariate and multivariate logistic regression analyses were used to identify independent risk factors for 28-day mortality, which were integrated into a nomogram prediction model. Discriminative ability, calibration, and clinical utility of the model were evaluated using the area under the receiver operating characteristic (ROC) curve (AUC), calibration curve, and decision curve analysis (DCA), respectively, and the model was compared with the APACHE II and SOFA scores.

**Results:**

Three independent risk factors were identified: number of dysfunctional organs (OR = 2.438, 95% CI: 1.634–3.638), septic shock (OR = 2.233, 95% CI: 1.034–4.822), and blood lactate level (OR = 1.207, 95% CI: 1.066–1.367). The 28-day mortality risk prediction model for patients with AIDS suffering from sepsis secondary to pulmonary infections in the ICU (MPASP) constructed with these factors exhibited an AUC of 0.877 (95% CI: 0.843–0.911), a sensitivity of 85.7%, and a specificity of 76.6% in the development cohort. In the temporal validation cohort, the AUC was 0.861 (95% CI: 0.778–0.943), with a sensitivity of 80.0% and a specificity of 82.5%. Calibration curves and DCA demonstrated good calibration and clinical utility. The MPASP has exhibited improved discriminative ability compared with the APACHE II score (AUC = 0.746) and SOFA score (AUC = 0.801) (both *P* < 0.01).

**Conclusions:**

The MPASP model, based on the number of dysfunctional organs, presence of septic shock, and blood lactate level reflecting the most severe early clinical status within the first 24 h of ICU admission, shows potential utility for predicting 28-day mortality in ICU-admitted AIDS patients with sepsis secondary to pulmonary infection. Simple and easy to apply, it may serve as a practical reference for clinical decision-making in managing this specific patient population.

## Background

Due to severe immune impairment, patients with AIDS are highly susceptible to various opportunistic infections, with pulmonary infections being the most common and severe complications [1–5]. Compared to the general population, opportunistic pulmonary infections remain a major threat to patients with AIDS [2, 4, 5]. Pulmonary infection not only significantly increases morbidity in patients with AIDS but also serves as a major driver for sepsis, leading to high morbidity and mortality [1–6]. Relevant studies have shown that among critically ill patients with AIDS admitted to the ICU (CD4⁺T cell < 200 cells/µL or complicated with opportunistic infections), pulmonary infection is an extremely prominent complication, with an incidence rate of 64%-70% [2, 5, 6]. Compared to patients without AIDS, those with AIDS are 2–3 times more likely to develop sepsis after pulmonary infection [1, 5–7]. When pulmonary infection progresses to sepsis, the mortality risk increases sharply, with a mortality rate of 47%-49% for uncomplicated sepsis and 61.5% for septic shock [2, 5]. Liu et al. [8] conducted a meta-analysis on sepsis in China, which revealed that the overall mortality rate of sepsis in the country stood at 29.0% and that of septic shock even reached 37.3%, both figures significantly higher than those observed in Europe and North America. Notably, epidemiological data on sepsis in high-risk populations remain particularly scarce. Therefore, developing an exclusive mortality prediction tool tailored to the immunocompromised subgroup of patients with AIDS holds considerable clinical significance for sepsis prevention and control in China.

To date, several mortality risk prediction models have been developed for general patients with sepsis, such as the acute physiology and chronic health evaluation II (APACHE II) and sequential organ failure assessment (SOFA). However, due to the unique immune status and disease characteristics of patients with AIDS, these models lack sufficient accuracy and specificity when predicting 28-day mortality in this subgroup with sepsis caused by pulmonary infections [9–12]. The immune function of patients with AIDS declines progressively, and their CD4⁺T lymphocyte counts continuously decrease, resulting in an infection response pattern distinct from that observed in the general population [9, 13]. Conventional prediction models fail to fully account for these specific factors. Therefore, this study aimed to develop a specific 28-day mortality risk prediction model for patients with AIDS suffering from sepsis secondary to pulmonary infections in the ICU. This model can help the early identification of high-risk patients, timely adjustment of treatment strategies, and rational allocation of medical resources to improve patients’ prognosis and reduce mortality.

## Methods

### Study design and setting

This was a retrospective, single-center observational study conducted at Chengdu Public Health Clinical Medical Center, A government-designated Grade A Tertiary infectious disease specialized hospital, primarily responsible for the diagnosis and treatment of infectious diseases including Mycobacterium tuberculosis infection and HIV infection. It focused on the development and validation of a 28-day mortality risk prediction model for patients with AIDS suffering from sepsis secondary to pulmonary infections in the ICU. The study included a model development cohort and a validation cohort. The development cohort was used for model training and internal validation, while the validation cohort was used for temporal validation. Patients enrolled from January 2016 to December 2023 were assigned to the development cohort, and those enrolled from January 2024 to June 2025 were assigned to the temporal validation cohort. The primary outcome was patient survival or death within 28 days of ICU admission (all-cause mortality). The study was reported following the Transparent Reporting of a Multivariable Prediction Model for Individual Prognosis or Diagnosis (TRIPOD) statement [14].

### Study population

The study population included patients with AIDS suffering from sepsis secondary to pulmonary infections admitted to the Department of Critical Care Medicine, Chengdu Public Health Clinical Medical Center from January 2016 to June 2025. The inclusion criteria were as follows: (1) age ≥ 18 years; and (2) concurrent diagnosis of pulmonary infection, sepsis, and AIDS at ICU admission. The diagnosis of pulmonary infection was made based on specific clinical symptoms, signs, chest imaging findings, and morphological identification or culture of pathogens from induced sputum, tracheal aspirate, or bronchoalveolar lavage fluid (when available) [15, 16]. Sepsis was diagnosed before ICU admission or within 24 h after admission, in line with the 2016 Sepsis-3 consensus definition [17], Patients admitted prior to the release of Sepsis-3 were retrospectively diagnosed based on medical records. The treatment of sepsis is guided by the core therapeutic principle encompassing source control of infection, early empirical anti-infective therapy and fluid resuscitation. The diagnosis of AIDS complied with the “Centers for Disease Control and Prevention (CDC) AIDS Surveillance Definition”. The exclusion criteria were as follows: (1) pregnant or lactating women; (2) ICU length of stay < 24 h; (3) voluntary withdrawal from ICU treatment; (4) With other primary infection sites outside the lungs; and (5) missing primary outcome data.

### Data collection

Patients with AIDS suffering from sepsis secondary to pulmonary infections typically present with severe conditions and rapid disease progression. Early prognosis prediction after ICU admission is crucial for timely treatment. To ensure clinical applicability and capture early clinical deterioration within the first 24 h of ICU admission, potential predictors were selected from routine examinations yielding results within this window. As potential model predictors, the following variables were retrospectively collected from electronic medical records: (1) demographic data (gender, age); (2) baseline clinical characteristics (heart rate, respiratory rate, mean arterial pressure, oxygenation index (PaO₂/FiO₂), number of dysfunctional organs (assessed by SOFA score), presence of multiple organ dysfunction syndrome (MODS), septic shock, and respiratory failure); (3) disease severity scores (APACHE II score, SOFA score); (4) laboratory test results (white blood cell count(WBC), neutrophil count, hemoglobin, platelet count, C-reactive protein(CRP), procalcitonin(PCT), blood urea nitrogen(BUN), serum creatinine(SCr), blood glucose, albumin, blood lactate, D-dimer, CD4⁺T cell count, and HIV viral load); (5) regular antiviral therapy before ICU admission; and (6) 28-day prognosis (survival/death) after ICU admission. 28-day prognosis data were obtained from discharge records, medical charts, or telephone follow-up. For variables with multiple test results within the first 24 h of ICU admission, the value most relevant to disease severity was selected, namely the one reflecting the most severe disease status during this early window, such as the maximum lactate level, the minimum oxygenation index (PaO₂/FiO₂), and the minimum platelet count. The number of dysfunctional organs was assessed using the SOFA score [18]: each organ system was scored 0–4 points based on dysfunction severity, with a score ≥ 2 defined as “sepsis-related organ dysfunction”. Based on the maximum SOFA score obtained within 24 h of the patients’ ICU admission, organ systems with a SOFA score ≥ 2 were enumerated as dysfunctional organs. In development cohort, the missing rate of alanine aminotransferase (ALT) data was relatively high (6.2%), with no statistically significant difference in ALT levels between the mortality and survival groups. Additionally, brain natriuretic peptide (BNP) was not included in routine testing due to restrictions imposed by medical insurance reimbursement policies, with valid test results available for less than 10% of patients. For these reasons, neither of the two indicators was included as a potential predictive factor.

### Missing data

In the development cohort, missing data rates were: 1.8% (7/385) for procalcitonin, 0.78% (3/385) for albumin, 0.78% (3/385) for CD4⁺T cell, 1.0% (4/385) for heart rate, and 1.3% (5/385) for respiratory rate. No missing data were detected for the 86 patients included in the validation cohort. In total, 22 cases had missing data, with all potential predictors having a missing rate < 2%. Variables with missing data were routine laboratory tests for patients with AIDS suffering from sepsis upon ICU admission, and the missing pattern was considered random. Comparison of baseline characteristics between 449 complete cases and 471 cohort cases revealed no significant differences; thus, complete case analysis was conducted for model development and validation.

### Statistical analysis

SPSS 29.0 and R 4.5.1 software were employed for data analysis. Categorical variables are expressed as counts (percentages), with intergroup comparisons conducted using the chi-squared test or Fisher’s exact test as appropriate. Continuous variables were tested for normality using the Shapiro-Wilk test. Normally distributed variables are expressed as mean ± standard deviation, and non-normally distributed variables are presented as median (interquartile range). Intergroup comparisons were conducted using the independent samples t-test and Mann-Whitney U test, respectively. The development cohort was divided into a death group and a survival group based on the primary outcome. Univariate analysis was conducted to compare intergroup differences. Variables with significant differences (*P* < 0.05) were analyzed for multicollinearity using the variance inflation factor (VIF). Independent variables with 1 ≤ VIF < 5 was included in a multivariate logistic regression analysis to screen independent risk factors for 28-day mortality. A visual nomogram prediction model was constructed by integrating independent risk factors. In the model development cohort and temporal validation cohort, the discriminative ability, calibration, and clinical utility of the model were evaluated using the AUC of the ROC curve, calibration curve, and DCA curve, respectively. Internal validation was conducted using 1000 repeated bootstrap samplings, and an AUC density plot was drawn to assess model stability. The Delong test was used to compare ROC curve differences between the nomogram, APACHE II score, and SOFA score. All statistical tests were two-tailed, with *P* < 0.05 deemed statistically significant.

**Fig. 1:**
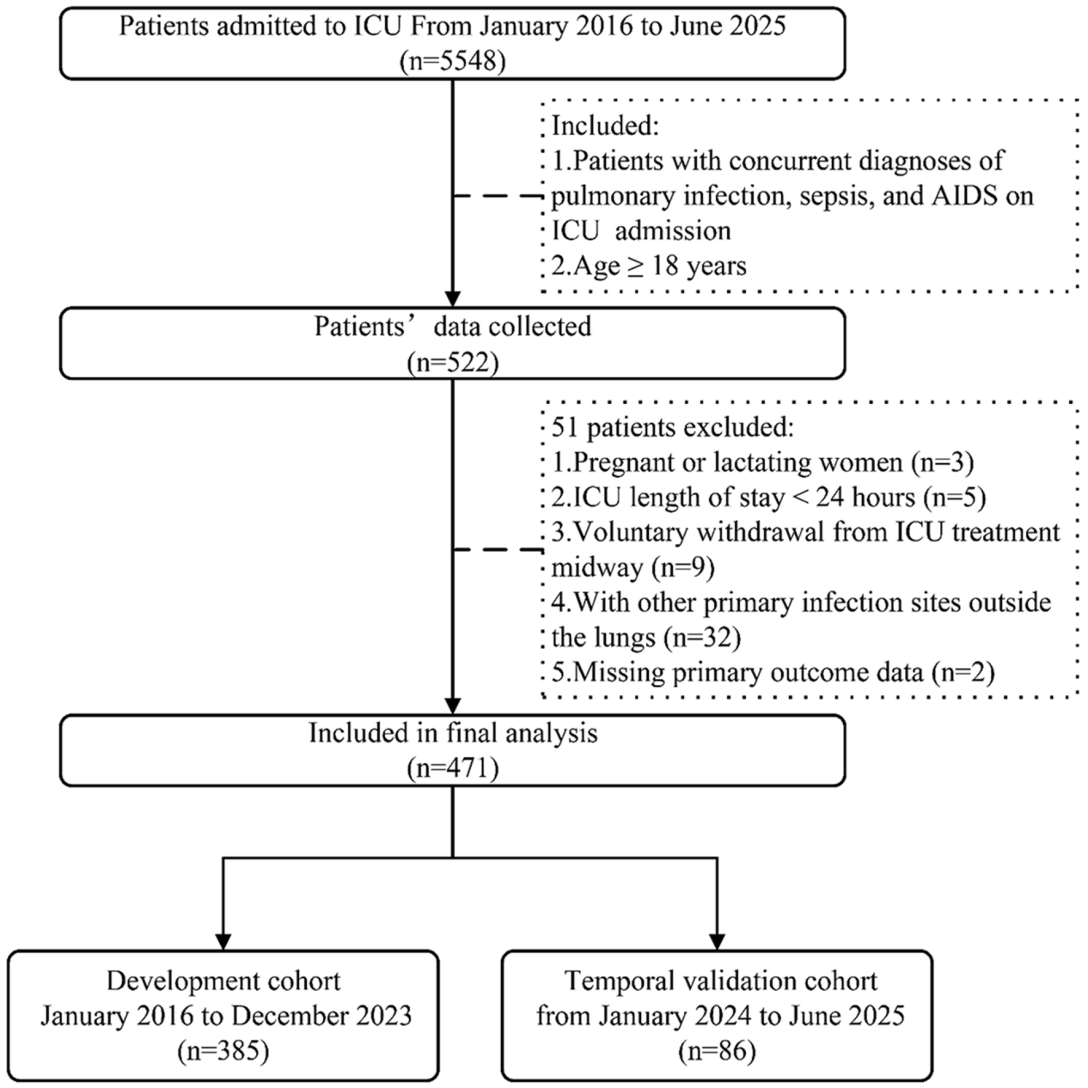
Flowchart illustrating the research process

## Results

### Clinical characteristics

As shown in Fig. 1, a total of 5548 critically ill patients were admitted to the ICU from January 2016 to June 2025, with 522 patients subsequently screened and enrolled in the present study in accordance with the inclusion criteria. Fifty-one patients were excluded based on the exclusion criteria: (1) pregnant or lactating women (*n* = 3); (2) ICU length of stay < 24 h (*n* = 5); (3) voluntary withdrawal from ICU treatment midway (*n* = 9); (4) With other primary infection sites outside the lungs. (*n* = 32); and (5) missing primary outcome data (*n* = 2). Finally, 471 patients were included in the study cohort, consisting of 385 in the development cohort and 86 in the temporal validation cohort. Males accounted for 81.31% (383/471) of the participants, with a mean age of 51.63 ± 15.34 years, and the 28-day mortality after ICU admission was 51.59% (243/471). At ICU admission, 77.10% (363/471) of patients suffered from respiratory failure; 60.29% (284/471) had septic shock; 92.78% (437/471) and 45.22% (213/471) suffered from 1 and ≥ 3 organ dysfunctions, with corresponding 28-day mortality rates of 57.67% (252/437) and 86.92% (186/213), respectively. The average APACHE II score and SOFA score were 22.04 and 7.55, respectively. There were no significant differences in baseline data between the development and temporal validation cohorts (*P* > 0.05) (Table 1), indicating good consistency.

**Table 1:** Comparison of baseline characteristics between the development cohort and the temporal validation cohort

Variables	Development cohort(*n* = 385)	Validation cohort(*n* = 86)	χ²/t/z	*P* value
**Demographics**				
Age, years, (mean ± SD)	51.4 ± 15.4	52.5 ± 15.1	-0.553	0.581
Gender			0.081	0.775
Female, n (%)	71(18.4)	17(19.8)		
Male, n (%)	314(81.6)	69(80.2)		
**Clinical Characteristics and Treatments**				
MODS			0.723	0.395
No, n (%)	302(78.4)	71(82.6)		
Yes, n (%)	83(21.6)	15(17.4)		
Septic shock			0.315	0.575
No, n (%)	160(41.7)	33(38.4)		
Yes, n (%)	224(58.3)	53(61.6)		
Respiratory Failure			0.615	0.433
No, n (%)	96(24.9)	18(20.9)		
Yes, n (%)	289(75.1)	68(79.1)		
Number of dysfunctional organs, median (Q1, Q3)	2(1,3)	2(2,3)	-1.555	0.120
APACHE II, (mean ± SD)	22.0 ± 8.2	22.2 ± 8.7	-0.149	0.882
SOFA, (mean ± SD)	7.5 ± 4.1	7.7 ± 4.2	-0.498	0.619
cART treatment			0.168	0.682
No, n (%)	196(51.0)	46(53.5)		
Yes, n (%)	188(48.9)	40(46.5)		
**Vital signs**				
Heart rate, times/min, (mean ± SD)	116.8 ± 24.6	114.0 ± 28.0	0.920	0.358
Respiratory rate, times/min, (mean ± SD)	32.1 ± 9.5	31.6 ± 8.4	0.475	0.635
MAP, mmHg, (mean ± SD)	87.1 ± 20.4	88.3 ± 19.4	-0.510	0.610
PaO₂/FiO₂, ratio, (mean ± SD)	183.7 ± 91.5	166.7 ± 87.8	1.566	0.118
**Laboratory data**				
Hemoglobin, g/L, median (Q1, Q3)	99.0(78.0,122.0)	96.0(71.0,113.0)	-1.088	0.276
HIV RNA, log_10_ copies/mL, (mean ± SD)	4.11 ± 1.77	4.11 ± 1.94	0.005	0.996
WBC count, ×10⁹/L, median (Q1, Q3)	6.84(3.72,11.38)	6.87(4.41,10.83)	-0.266	0.790
Neutrophil Count, ×10⁹/L, median (Q1, Q3)	6.02(2.93,10.41)	5.96(3.79,9.87)	-0.320	0.749
Platelet Count, ×10⁹/L, median (Q1, Q3)	123.0(59.0,201.5)	132.5(57.8,210.0)	-0.483	0.629
CRP, mg/L, median (Q1, Q3)	86.8(37.1,153.5)	97.7(45.6,154.2)	-0.702	0.483
PCT, ng/mL, median (Q1, Q3)	0.75(0.19,5.39)	1.21(0.27,4.64)	-0.487	0.626
BUN, mmol/L, median (Q1, Q3)	7.5(5.2,13.0)	7.5(5.1,12.6)	-0.468	0.640
SCr, µmol/L, median (Q1, Q3)	74.0(52.0,138.8)	75.4(51.5,117.3)	-0.085	0.933
Blood glucose, mmol/L, median (Q1, Q3)	6.6(5.2,8.5)	6.6(5.4,8.9)	-0.656	0.512
Serum albumin, g/L, median (Q1, Q3)	26.3(22.2,30.4)	25.8(21.4,28.8)	-1.578	0.115
Lactate, mmol/L, median (Q1, Q3)	2.4(1.4,4.6)	2.4(1.6,4.2)	-0.057	0.955
D-Dimer, µg/mL, median (Q1, Q3)	4.0(1.8,8.7)	5.4(2.2,8.9)	-1.576	0.115
CD4^+^T, cells/µL, median (Q1, Q3)	31.0(12.0,95.0)	26.0(9.0,120.0)	-0.055	0.957
28-day mortality, n (%)	197(51.2)	46(53.5)	-0.390	0.700

Note: CD4^+^T: CD4-positive T lymphocyte; MODS: multiple organ dysfunction syndrome; cART: combination antiretroviral therapies; APACHE II: acute physiology and chronic health evaluation II; SOFA: sequential organ failure assessment; MAP: mean arterial pressure; PaO_2_: partial pressure of arterial oxygen; FiO_2_, inspired oxygen fraction; HIV: human immunodeficiency virus; WBC: white blood cell; CRP: C-reactive protein; PCT: procalcitonin; BUN: blood urea nitrogen; SCr: serum creatinine

### Predictor screening and construction of the nomogram model

Univariate analysis identified significant differences (*P*<0.05) between the death and survival groups in the development cohort for the following variables (Table 2): septic shock, respiratory failure, MODS, APACHE II score, SOFA score, heart rate, number of dysfunctional organs, platelet count, C-reactive protein, procalcitonin, blood urea nitrogen, serum creatinine, albumin, blood lactate, and D-dimer. VIF analysis of these variables revealed a maximum VIF of 3.53 (blood urea nitrogen) and a minimum of 1.15 (respiratory failure), indicating weak multicollinearity. All variables were included in the multivariate analysis. Multivariate logistic regression analysis indicated that the number of dysfunctional organs (*P*<0.001, OR = 2.438, 95% CI: 1.634–3.638), septic shock (*P* = 0.041, OR = 2.233, 95% CI: 1.034–4.822), and blood lactate level (*P* = 0.003, OR = 1.207, 95% CI: 1.066–1.367) were independent risk factors for 28-day mortality (Table 3).

**Table 2:** Univariate analysis of baseline data between survivors and non-survivors in the model development cohort

Variables	Survivors (*n* = 188)	Non-survivors (*n* = 197)	χ²/t/z	*P* value
**Demographics**				
Age, years, (mean ± SD)	50.4 ± 14.4	52.4 ± 16.3	-1.239	0.216
Gender			3.713	0.054
Female, n (%)	42(22.3)	29(14.7)		
Male, n (%)	146(77.7)	168(85.3)		
**Clinical Characteristics and Treatments**				
MODS			57.296	< 0.001
No, n (%)	178(94.7)	124(62.9)		
Yes, n (%)	10(5.3)	73(37.1)		
Septic shock			89.415	< 0.001
No, n (%)	124(65.9)	36(18.4)		
Yes, n (%)	64(34.0)	160(81.6)		
Respiratory failure			27.179	< 0.001
No, n (%)	69(36.7)	27(13.7)		
Yes, n (%)	119(63.3)	170(86.3)		
Number of dysfunctional organs, median (Q1, Q3)	1(1,2)	3(2,4)	-11.975	< 0.001
APACHE II, (mean ± SD)	18.4 ± 5.5	25.5 ± 8.8	-9.526	< 0.001
SOFA, (mean ± SD)	5.4 ± 2.7	9.5 ± 4.2	-11.451	< 0.001
cART treatment			2.641	0.104
No, n (%)	88(46.8)	108(55.1)		
Yes, n (%)	100(53.2)	88(44.9)		
**Vital signs**				
Heart rate, times/min, (mean ± SD)	110.8 ± 22.4	122.5 ± 25.3	-4.783	< 0.001
Respiratory rate, times/min, (mean ± SD)	31.1 ± 9.2	33.0 ± 9.8	-1.955	0.051
MAP, mmHg, (mean ± SD)	87.8 ± 18.7	86.5 ± 21.9	0.631	0.528
PaO₂/FiO₂, ratio, (mean ± SD)	188.7 ± 94.5	178.9 ± 88.5	1.050	0.295
**Laboratory data**				
Hemoglobin, g/L, median (Q1, Q3)	102.0(79.0,123.0)	96.0(74.0,119.0)	-1.874	0.061
HIV RNA, log_10_ copies/mL, (mean ± SD)	4.13 ± 1.80	4.08 ± 1.74	0.265	0.791
WBC count, ×10⁹/L, median (Q1, Q3)	6.81(3.72,11.27)	6.90(3.44,11.82)	-0.057	0.955
Neutrophil count, ×10⁹/L, median (Q1, Q3)	5.86(2.99,10.29)	6.28(2.89,10.54)	-0.113	0.91
Platelet count, ×10⁹/L, median (Q1, Q3)	144.5(73.3,237.8)	99.0(50.0,171.0)	-4.537	< 0.001
CRP, mg/L, median (Q1, Q3)	69.6(25.9,123.6)	107.4(43.9,168.0)	-3.551	< 0.001
PCT, ng/mL, median (Q1, Q3)	0.43(0.15,1.97)	1.98(0.37,11.50)	-5.658	< 0.001
BUN, mmol/L, median (Q1, Q3)	6.3(4.8,9.5)	9.3(6.1,15.7)	-5.152	< 0.001
SCr, µmol/L, median (Q1, Q3)	65.2(50.0,94.9)	85.5(56.0,168.8)	-4.185	< 0.001
Blood glucose, mmol/L, median (Q1, Q3)	6.5(5.3,8.1)	6.7(5.2,9.4)	-1.183	0.237
Serum albumin, g/L, median (Q1, Q3)	27.7(23.3,31.3)	25.2(21.4,29.6)	-3.554	< 0.001
Lactate, mmol/L, median (Q1, Q3)	1.7(0.9,2.7)	3.6(2.2,6.5)	-9.449	< 0.001
D-Dimer, µg/mL, median (Q1, Q3)	2.9(1.3,5.4)	5.5(3.2,11.5)	-6.430	< 0.001
CD4^+^T, cells/µL, median (Q1, Q3)	31.0(12.0,104.3)	32.0(11.0,89.5)	-0.636	0.525

Note: Abbreviations as in Table 1

**Table 3:** Multivariate logistic regression analysis of 28-day mortality risk in ICU-admitted patients with sepsis secondary to pulmonary infections

Variables	Β	SE	z	*P*	OR	95% CI
Septic shock	0.803	0.393	2.045	0.041	2.233	1.034 ~ 4.822
Number of dysfunctional organs	0.891	0.204	4.363	< 0.001	2.438	1.634 ~ 3.638
Lactate	0.189	0.063	2.969	0.003	1.207	1.066 ~ 1.367
MODS	-0.401	0.560	-0.716	0.474	0.669	0.223 ~ 2.008
Respiratory failure	0.605	0.392	1.542	0.123	1.831	0.849 ~ 3.948
APACHE II	-0.003	0.029	-0.113	0.910	0.997	0.941 ~ 1.056
SOFA	0.091	0.066	1.367	0.172	1.095	0.961 ~ 1.247
Heart rate	0.009	0.007	1.226	0.220	1.009	0.995 ~ 1.023
Platelet count	-0.001	0.002	-0.752	0.452	0.999	0.995 ~ 1.002
CRP	0.003	0.002	1.492	0.136	1.003	0.999 ~ 1.007
PCT	-0.010	0.006	-1.560	0.119	0.990	0.978 ~ 1.003
BUN	0.027	0.025	1.071	0.284	1.027	0.978 ~ 1.080
SCr	0.001	0.001	0.818	0.414	1.001	0.999 ~ 1.004
Serum albumin	0.016	0.018	0.886	0.376	1.016	0.981 ~ 1.053
D-Dimer	0.001	0.017	0.031	0.975	1.001	0.967 ~ 1.035

Note: Abbreviations as in Table 1

**Fig. 2:**
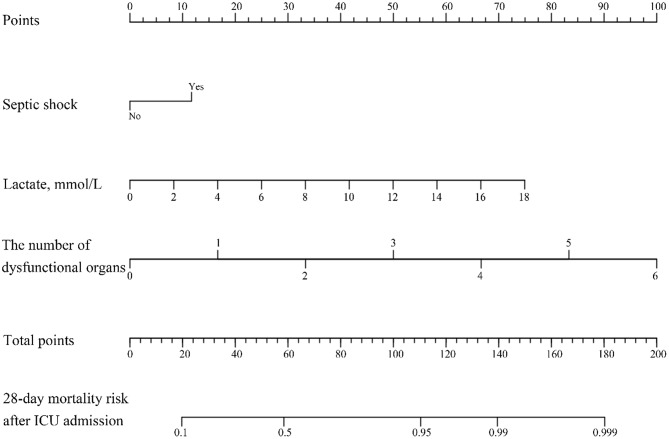
The nomogram model for predicting 28-day mortality risk in patients with AIDS suffering from sepsis secondary to pulmonary infections in the ICU

Combining these three independent risk factors, we constructed a visual nomogram prediction model (Fig. 2), namely the 28-Day Mortality Risk Prediction Model for Patients with AIDS Suffering from Sepsis Secondary to Pulmonary Infections in the ICU (MPASP). The model usage is as follows: Step 1: Locate predictor values – First, identify the patient’s values for each predictor on the corresponding axes. For example, consider a patient with the following indicators: Septic shock = Yes; Lactate = 1.55 mmol/L; The number of dysfunctional organs = 4. Locate these values on their respective axes; Step 2: Obtain individual item scores – Draw a vertical line upward from each value to the “Points” axis to get the score for each item. For this patient: the “Septic shock (Yes)” position corresponds to 10 points; “Lactate (1.55 mmol/L)” corresponds to 7.5 points; “The number of dysfunctional organs (4)” corresponds to 66 points; Step 3: Calculate total score – Sum the individual scores to get the total score (Total Points). For this patient, the total score is calculated as 10 + 7.5 + 66 = 83.5; Step 4: Determine predicted mortality – Draw a vertical line downward from the total score on the “Total Points” axis to intersect with the outcome axis (28-day mortality risk after ICU admission). The intersection value represents the patient’s predicted 28-day mortality probability. For this patient, the total score of 83.5 corresponds to a predicted 28-day mortality probability of 0.71(71%).

### Model performance evaluation

**Fig. 3:**
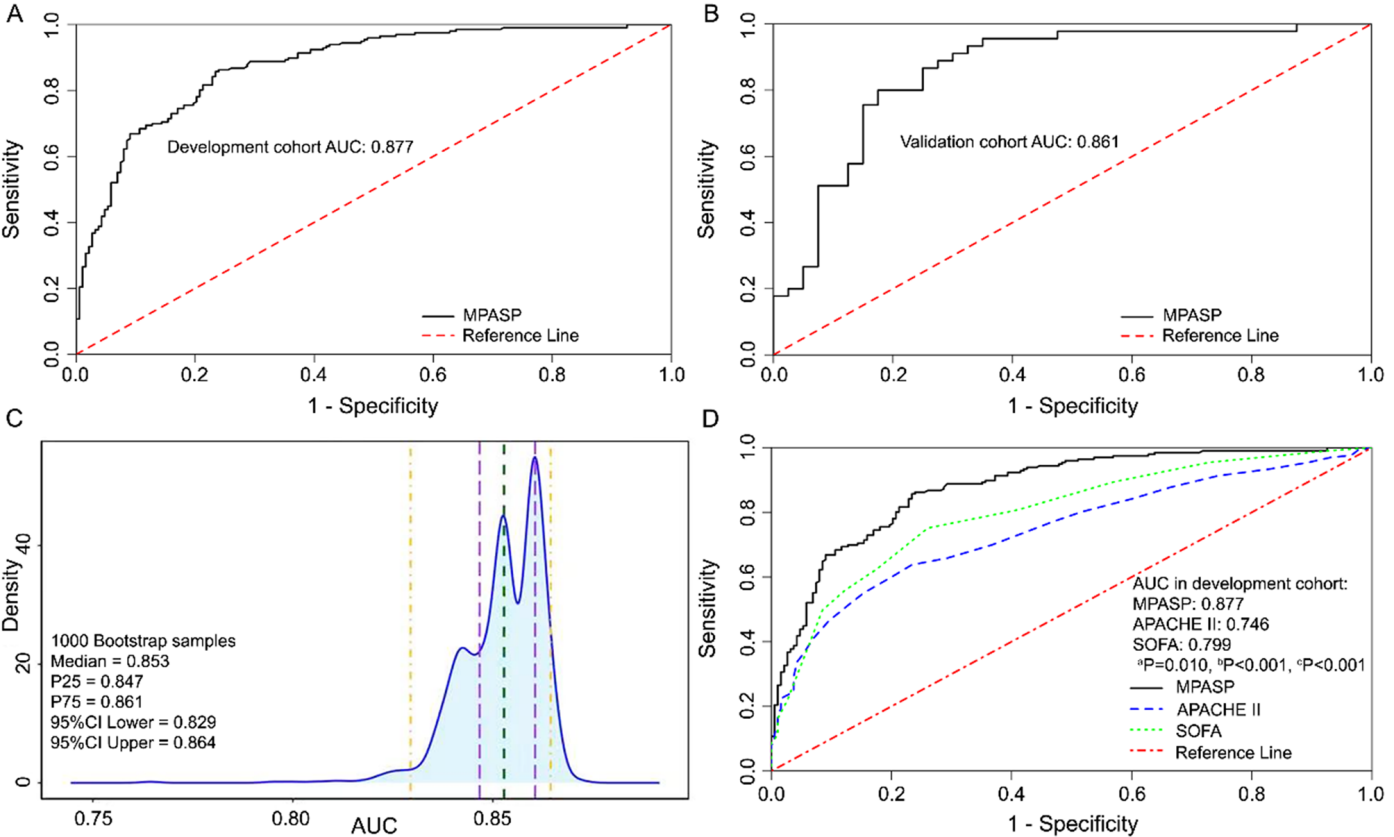
The discriminability of the MPASP. **A** and **B**, ROC curves for internal and temporal validation of MPASP, A: internal validation; **B:** temporal validation; C: AUC density distribution of 1000 times bootstrap sampling; **D**: The ROC curves of the MPASP, APACHE II, and SOFA in the development cohort. MPASP: 28-day mortality risk prediction model for patients with AIDS suffering from sepsis secondary to pulmonary infections in the ICU; ROC: receiver operating characteristic; AUC: area under the ROC curve; APACHE II: acute physiology and chronic health evaluation II; SOFA: sequential organ failure assessment. aP: APACHE II vs. SOFA; bP: APACHE II vs. MPASP; cP: MPASP vs. SOFA

**Fig. 4:**
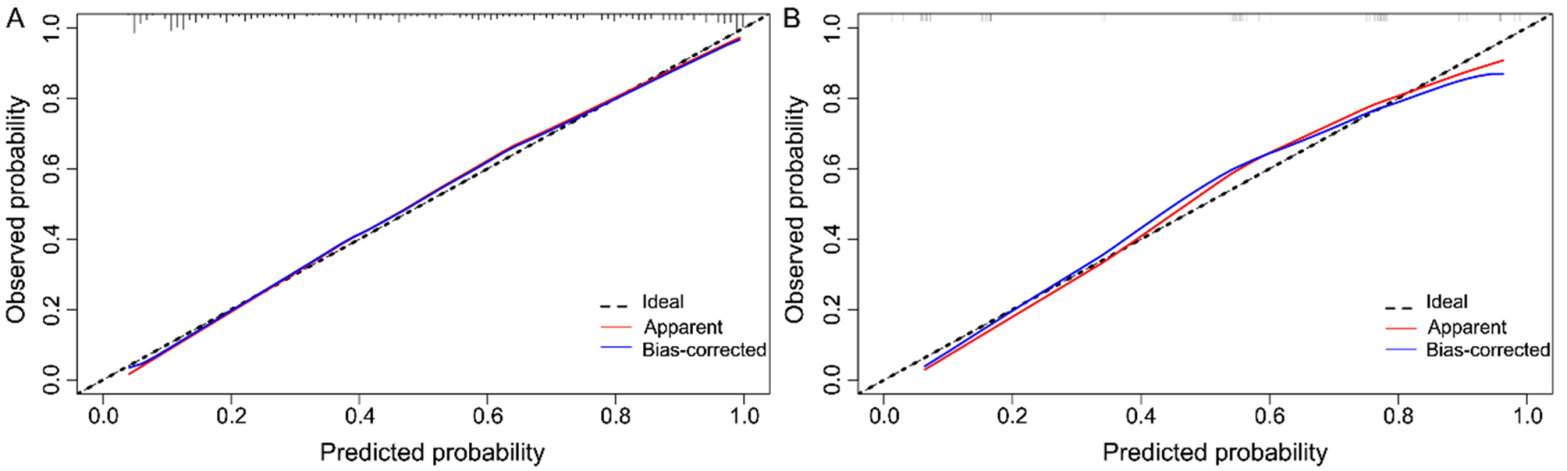
Calibration curves of the MPASP. **A**: Training set, **B**: Validation set. The calibration of this model is based on the agreement between the predicted and observed probability of 28-day mortality in patients with AIDS suffering from sepsis secondary to pulmonary infections in the ICU

In the development cohort, the overall AUC of the MPASP model was 0.877 (95% CI: 0.843–0.911) (Fig. 3A), with a sensitivity of 85.7% and specificity of 76.6%, indicating good discriminative ability. The calibration curve (Fig. 4A) indicated that the predicted probability of the model was roughly concordant with the observed probability. Only at extremely high or low predicted probabilities, the model’s prediction was slightly lower than the actual probability, which aligned with the ideal prediction curve after bias correction, suggesting good calibration.

### Model validation

**Fig. 5:**
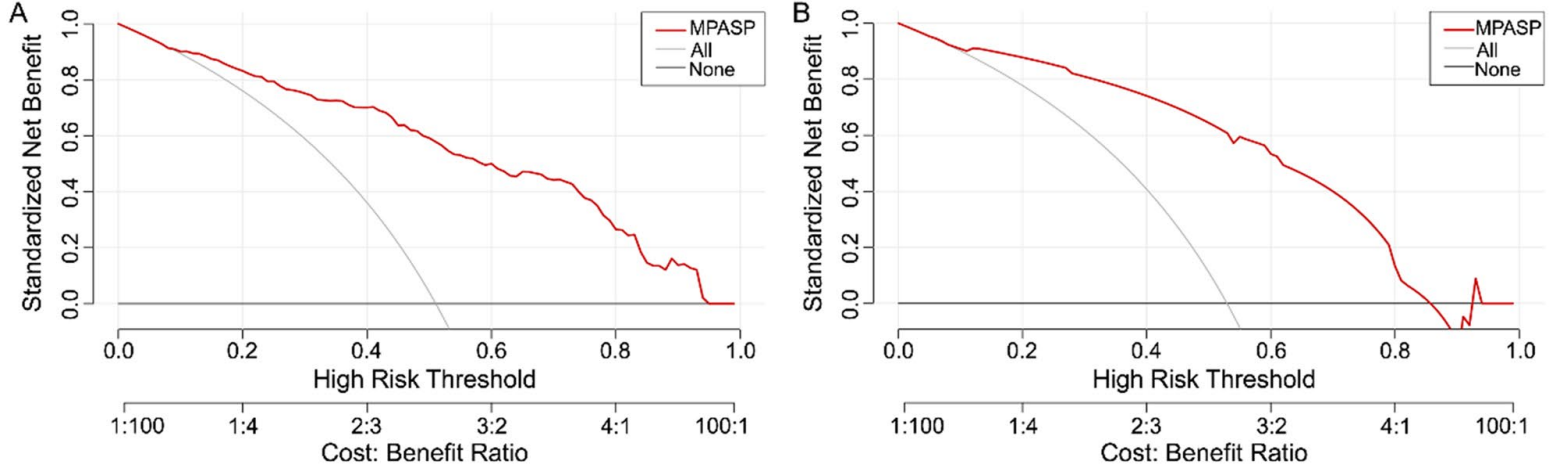
The DCA curves of the MPASP in patients with AIDS suffering from sepsis secondary to pulmonary infections in the ICU. **A**: Training set, **B**: Validation set

The AUC density distribution plot from internal validation (Fig. 3C) showed a median AUC of 0.853 (95% CI: 0.829–0.864) after 1000 bootstrap samplings, with an interquartile range of 0.014, which was consistent with a medium-high discriminative range. The median AUC of internal validation was slightly lower than that of the full development cohort, likely due to the optimistic bias of the full-cohort AUC. Internal validation confirmed good discriminative ability and stability of MPASP. In temporal validation, the AUC of the model was 0.861 (95% CI: 0.778–0.943) (Fig. 3B), with a sensitivity of 80.0% and a specificity of 82.5%. The calibration curve (Fig. 4B) also confirmed good calibration. DCAs were plotted for both cohorts to assess the clinical utility of MPASP (Fig. 5).

### Model comparison: MPASP vs. SOFA and APACHE II Scores (ROC Comparison)

To evaluate the effectiveness of the model, we plotted ROC curves of MPASP, SOFA, and APACHE II scores in the development cohort (Fig. 3D), obtaining AUC values of 0.877, 0.801, and 0.746, respectively. The Delong test revealed that the AUC of MPASP was significantly higher than that of SOFA (*P*<0.001) and APACHE II (*P*<0.001) (Table 4).

**Table 4:** Comparison of ROC curves for MPASP, SOFA, and APACHE II in the model development cohort

Predictive model	AUC	95% CI	Sensitivity	Specificity	Best threshold	*P* value
APACHE II	0.746	0.698 ~ 0.795	0.638	0.766	21.000	^a^*P* = 0.010
SOFA	0.801	0.757 ~ 0.845	0.755	0.739	6.000	^b^*P*<0.001
MPASP	0.877	0.843 ~ 0.911	0.857	0.766	0.433	^c^*P* < 0.001

Note: ^a^P: APACHE II vs. SOFA; ^b^P: APACHE II vs. MPASP; ^c^P: MPASP vs. SOFA. MPASP: 28-day mortality risk prediction model for patients with AIDS suffering from sepsis secondary to pulmonary infections in the ICU

## Discussion

In this study, we developed and validated the MPASP model to predict 28-day mortality among ICU-admitted patients with AIDS suffering from sepsis secondary to pulmonary infection. The model is built on three independent risk factors—the number of dysfunctional organs, presence of septic shock, and blood lactate level—for which values were captured during the first 24 h of ICU admission, reflecting early clinical deterioration within this window. The MPASP model exhibited excellent discriminative ability (AUC > 0.8) [19] and good calibration in both the development and temporal validation cohorts. Furthermore, the model demonstrated statistically significant improved discriminative ability for the primary study outcome (28-day mortality) compared with the APACHE II and SOFA scores in the development cohort, as indicated by a higher AUC value (Delong test, all *P* < 0.01). Decision curve analysis (DCA; Fig. 5) revealed that the MPASP model generated a positive net benefit (above the “None” curve) and outperformed the “All-intervention” strategy (above the “All” curve) across the clinically relevant 0.2–0.9 threshold range [20], The MPASP model is a dedicated prediction tool developed for the specific population of AIDS patients with sepsis secondary to pulmonary infection, and it is constructed based on routine clinical data obtained during the first 24-hour window after ICU admission. With its simplicity and ease to apply, this model may provide a practical reference for the early risk stratification of patients and clinical management decision-making in clinical practice.

Decision curve analysis (DCA) was performed to assess the clinical utility of the MPASP model in the study population (Fig. 5A and B). The x-axis represents the threshold probability of 28-day mortality, and the y-axis quantifies the model’s net clinical benefit relative to the two extreme clinical strategies of universal intervention (for all patients) and no intervention (for any patient). DCA results confirmed that the MPASP model generated a sustained positive net clinical benefit for 28-day mortality prediction across the clinically relevant threshold probability range of 0.2–0.9, with its net benefit remaining consistently higher than that of both extreme strategies throughout this interval. This key finding validates the practical clinical utility of the MPASP model and provides a quantitative foundation for clinical risk stratification in ICU-admitted patients with AIDS complicated by sepsis secondary to pulmonary infection. Specifically, the DCA-defined threshold range enables stratification of patients into distinct risk groups based on their predicted 28-day mortality probability: patients with a predicted mortality probability at the lower end of the 0.2–0.9 range are categorized as low-to-moderate risk; patients with a predicted mortality probability at the higher end of the 0.2–0.9 range are classified as high risk. This translation of threshold probabilities into clinical decision-making in the ICU via risk stratification maximizes the MPASP model’s value as an adjunct to clinical judgment and helps mitigate the potential for over-intervention or under-intervention in the ICU management of this specific patient population.

This study identified the number of dysfunctional organs as an independent risk factor for 28-day mortality (OR = 2.438, 95% CI: 1.634–3.638). The 28-day mortality of patients with ≥ 3 organ dysfunctions reached 86.9%, which was significantly higher than that of patients with single organ dysfunction (57.6%). This aligns with the core pathological mechanism of sepsis: uncontrolled infection-induced systemic inflammatory response (SIRS) progressively impairs organ function [17], while patients with AIDS have impaired immune clearance capacity, leading to faster infection spread and more severe organ damage [2, 21]. Notably, organ damage in patients with AIDS may involve both “direct infection-induced damage” and “indirect aggravation by immune deficiency”. Respiratory failure caused by pulmonary infection can rapidly progress to MODS, and patients with low abundance of CD4⁺T cell are more prone to immune paralysis, failing to control infection spread [22]. By incorporating the number of dysfunctional organs as a core predictor, the model indirectly reflects the immune status and infection severity in patients with AIDS.

Septic shock was an independent risk factor (OR = 2.233, 95% CI: 1.034–4.822) in this study, highly consistent with the clinical characteristics of sepsis in patients with HIV. Immune deficiency accelerates the progression of infection to septic shock, and responsiveness to vasoactive drugs may remain poorer. Additionally, HIV-related chronic inflammation (e.g., endothelial cell activation, coagulation dysfunction) may aggravate tissue hypoperfusion after shock [7]. The inclusion of shock in the model enables rapid identification of high-risk individuals with “infection-induced circulatory failure”, providing a basis for early fluid resuscitation and treatment with vasoactive drugs.

The blood level of lactate (OR = 1.207, 95% CI: 1.066–1.367) is a classic indicator of tissue hypoxia and metabolic disorders [23], with important predictive value in AIDS-associated sepsis. Several studies have confirmed that blood lactate is an independent risk factor for 28-day mortality in patients with AIDS suffering from sepsis [16, 24–26], which is consistent with our findings. In patients with AIDS, elevated blood levels of lactate may result not only from shock-related tissue hypoperfusion but also from uncontrolled infection due to immune deficiency (e.g., metabolic disorders caused by excessive bacterial load) [22, 24, 25]. Due to the decreased number of CD4⁺T cell, pulmonary infection in patients with HIV can easily lead to sepsis. Bacterial toxins activate inflammatory responses, causing vasodilation and microcirculatory disorders. Impaired mitochondrial function in monocytes reduces lactate clearance, finally exacerbating lactate accumulation [27]. As a rapidly detectable indicator within 24 h, blood lactate level can indicate “occult tissue hypoxia” in the early stages, especially in patients without typical symptoms of shock, aiding early risk identification.

In this study, neither HIV RNA levels nor CD4^+^ T cell counts were predictive of 28-day mortality, this finding consistent with the results of multiple studies [26, 28, 29]. Among ICU-admitted AIDS patients with sepsis secondary to pulmonary infection, the 28-day mortality risk is more closely associated with critical illness factors—specifically septic shock, blood lactate levels, and the number of dysfunctional organs—rather than HIV/AIDS-related phenotypic characteristics, such as CD4^+^ T cell counts and HIV RNA levels. A study conducted in China that focus on predict In-hospital mortality in HIV/AIDS patients with pneumocystis pneumonia identified a CD4^+^ T cell count of less than 50 cells/µL as a significant predictor of In-hospital mortality [15], However, the population included in the study were not patients with sepsis. This discrepancy in study findings may be attributed to three key factors. Firstly, mortality in AIDS patients with sepsis is a direct consequence of the acute pathological process of sepsis, which is predominantly driven by clinical indicators reflecting the severity of acute infection rather than chronic HIV-related immune impairment [26, 27]. Secondly, as an acute life-threatening infection, sepsis can cause severe acute immune dysregulation in the host, manifested by significant reduction in CD4^+^ T cells, loss of core effector functions, and imbalance of immune subgroups. The CD4^+^ T cell count measured during the acute phase of sepsis mainly reflects the transient immune stress state caused by severe infection, rather than the potential degree of immune deficiency related to AIDS in the patient’s body [30, 31]. Additionally, the widespread clinical implementation of antiretroviral therapy (ART) for HIV infection, the constraints of the short 28-day study time window for mortality assessment, and the masking effect of various clinical confounding factors collectively contribute to the loss of clinical significance of HIV RNA levels and CD4^+^ T cell counts as risk factors for mortality in this specific patient population. A six-month survival rate study on severely ill patients with AIDS-related diseases and tuberculosis found that the 6-month mortality rate of the patients was closely related to a minimum CD4^+^ T cell count of less than 50 cells/µL [32]. This is likely because HIV/AIDS-related phenotypic characteristics are generally associated with mortality in patients with severe AIDS, but they primarily reflect patients’ long-term immune baseline and susceptibility to infections, and thus correlate with patients’ long-term prognosis. When sepsis develops, this association has relatively weak independent predictive value for patients’ short-term (e.g., 28-day) survival, as it is overshadowed by the severity of sepsis itself.

In comparison with other analogous studies, the predictive factors identified in the present study—number of dysfunctional organs, septic shock, and blood lactate level—have all been validated to possess prognostic value for short-term mortality risk in patients with sepsis in previous research [24, 28, 33, 34]. The key distinction lies in the focus of this study on a specific subgroup: ICU-admitted patients with AIDS complicated by sepsis secondary to pulmonary infection, who face an elevated risk of short-term mortality. To the best of our knowledge, the MPASP prediction model developed in this study is the first to be tailored for the 28-day mortality risk assessment of this particular population.

The MPASP model demonstrated improved discriminative performance for 28-day mortality prediction in this specific population relative to the APACHE II and SOFA scores, primarily because MPASP was specifically developed for patients with AIDS suffering from sepsis secondary to pulmonary infections in the ICU. It fully accounts for their unique immune status and disease characteristics. APACHE II has inherent limitations in its adaptability to HIV-related immune deficiency when assessing 28-day mortality risk in this population. Traditional physiological indicators, such as body temperature and white blood cell count, are prone to false negative results due to HIV-related immune paralysis. Cribbs et al. showed that the abnormal values of these indicators in patients with HIV and concomitant sepsis were only 28%, much lower than that in the general population with sepsis [25]. Although SOFA can assess the severity of organ failure, it does not integrate the prognostic value of shock and blood lactate, failing to capture metabolic disorders and circulatory risks caused by early infection in patients with HIV. Especially, they show insufficient efficacy in risk stratification within 24 h of admission [15, 35–38]. Wei et al. [39]developed a nomogram for elderly ICU-admitted patients with urosepsis, and its area under the receiver operating characteristic curve (AUC) of 0.789 was significantly higher than those of the APACHE II (AUC = 0.656) and SOFA (AUC = 0.735). This finding confirms that traditional scoring systems struggle to capture the pathophysiological characteristics of specific populations. Analogously, the MPASP model in the present study exhibited strong adaptability for patients with AIDS, and its AUC (0.877) was substantially superior to those of APACHE II (0.746) and SOFA (0.801). This further validates the clinical value of the principle that “special populations need exclusive predictive tools.”

## Limitations

This study had several limitations: (1) The single-center retrospective design may inherently introduce selection bias and information bias. Derived from a specialized infectious disease hospital, the study cohort may have distinct characteristics (e.g., a high proportion of patients with severe immunodeficiency with a median CD4⁺T cell count < 30 cells/µL; a pathogen spectrum dominated by opportunistic infections) compared to general hospitals, which may limit the external generalizability of the model [13]; (2) This study excluded 9 patients who voluntarily withdrew from ICU treatment and 32 patients with concurrent non-pulmonary primary infections. Such exclusions may reduce cohort heterogeneity and introduce selection bias—these patients are common in real-world ICU settings, and their exclusion may restrict the model’s applicability to a relatively “uncomplicated” subgroup; (3) The temporal validation cohort is relatively small (*n* = 86), which may compromise the stability of AUC estimates and thereby weaken the reliability of model performance validation results; (4) Only logistic regression was used for model development, without performance comparison with machine learning algorithms (e.g., random forest, extreme gradient boosting [XGBoost]). This may miss opportunities to further improve predictive efficacy, especially with an expanded sample size; (5) The outcome assessment is incomplete. This study only focused on 28-day all-cause mortality and did not explore long-term outcomes such as 90-day or 1-year mortality; (6) Etiological data were not included in the potential predictive factors, which may lead to the miss of more valuable predictors.

To address the aforementioned limitations and enhance the rigor, generalizability, and clinical value of this study, future multicenter prospective observational cohort studies are needed to validate our findings. Additionally, the scope of outcome assessment should be expanded from 28-day mortality to long-term mortality (90-day and 1-year) and non-mortality outcomes (e.g., length of ICU stays, duration of mechanical ventilation, organ function recovery time, and ART resumption status) to more comprehensively evaluate sepsis-related prognosis. Furthermore, by transforming the optimized model into practical tools, such as simplified scoring sheets and mobile applications, we can compare patient outcomes (e.g., antibiotic initiation time, mortality) between clinicians who use this tool and those who adopt conventional clinical tools. This can help verify the clinical utility of this model and provide support for practical clinical decision-making.

## Conclusion

The MPASP model, constructed based on the number of dysfunctional organs, presence of septic shock, and blood lactate level reflecting the most severe early clinical status within the first 24 h of ICU admission, demonstrates potential utility for predicting 28-day mortality in ICU-admitted patients with AIDS complicated by sepsis secondary to pulmonary infection. With its simplicity and ease of application, this model may offer a practical reference to assist in clinical decision-making for the management of this specific patient population.

## Data Availability

The datasets utilized and/or analyzed in the present study can be obtained from the corresponding author upon reasonable request.

## Abbreviations

AIDS: Acquired Immunodeficiency Syndrome
ROC: Receiver Operating Characteristic
AUC: Area Under the ROC Curve
APACHE II: Acute Physiology and Chronic Health Evaluation II
SOFA: Sequential Organ Failure Assessment
MPASP: 28-day mortality risk prediction model for patients with aids suffering from sepsis secondary to pulmonary infections in the ICU
ICU: Intensive Care Unit
DCA: Decision Curve Analysis
CD4+T: CD4-Positive T Lymphocyte
MODS: Multiple Organ Dysfunction Syndrome
CI: Confidence interval
OR: Odds ratio
